# LIGHT POLLUTION: Light at Night and Breast Cancer Risk Worldwide

**DOI:** 10.1289/ehp.118-a525

**Published:** 2010-12

**Authors:** Angela Spivey

**Affiliations:** **Angela Spivey** writes from North Carolina about science, medicine, and higher education. She has written for *EHP* since 2001 and is a member of the National Association of Science Writers

Several studies over the last decade have suggested that the modern practice of keeping our bodies exposed to artificial light at night, or LAN, increases cancer risk, especially for cancers (such as breast and prostate cancers) that require hormones to grow. Women who work night shifts have shown higher rates of breast cancer,^1^ whereas blind women, who are not likely to be exposed to or perceive LAN, have shown decreased risks.^2^ In 2007, the International Agency for Cancer Research declared shiftwork a probable human carcinogen.^3^ Now a large study of 164 countries adds another piece of evidence, implicating overall light pollution.

The study, conducted by University of Connecticut epidemiologist Richard Stevens and colleagues at the University of Haifa, showed that higher population-weighted country-level LAN levels were associated with higher incidence of breast cancer.^4^ A sensitivity test indicated a 30–50% increased risk of breast cancer in countries with the highest versus lowest LAN levels. No such association was found between LAN and incidence of non-hormone-dependent lung, colorectal, larynx, or liver cancers in women.

“We took the top-level view and said, ‘If there really is causation going on, LAN levels worldwide should correlate well with breast cancer incidence,’” Stevens says. “This is a necessary but not sufficient condition for a potentially large effect. If we had seen no relationship between country LAN level and breast cancer risk, that would have been good evidence against a large effect of LAN on breast cancer risk.”

Tulane University cancer biologist David Blask points out the implications go beyond shiftwork. “This study suggests that all of us who live in industrialized society have the potential to have our circadian system disrupted by too much light at night, and this risk is potentially not restricted to a smaller percentage of the population that is exposed because of their occupation,” Blask says.

Harvard epidemiologist Eva Schernhammer agrees that the positive result from this study adds more evidence to the idea that LAN exposure contributes to breast cancer risk. But as an ecological study,^5^ even if the result had been negative, it would not be strong enough to rule out evidence from prior case–control studies, she says.

The study authors point out that because of the ecological nature of the study, it did not control for behavior that would reduce individuals’ exposure to LAN, such as sleeping. If people are actually asleep, then little to no light would reach their retinas, Stevens says, adding, “Three of four good prospective studies have reported a lower risk of breast cancer in women who report a long sleep duration.”^6^ Stevens thinks of reported sleep duration as a surrogate for time spent in the dark. But people do wake in the middle of the night, he points out, and even brief periods of open eyes during the night could expose the retina to LAN.

The new study highlights the need to understand the mechanisms behind the association between cancer and LAN, which aren’t clear, Stevens says. Previously, Blask and colleagues famously showed that a key factor in the connection is melatonin, a hormone produced in nighttime darkness that promotes sleep.^7^ They showed that growth and metabolism of human breast cancers growing in rats slowed when the tumors were perfused with melatonin-rich human blood collected during the night. In contrast, growth and metabolism were unchanged in tumors perfused with blood in which melatonin levels had been suppressed because of even a brief LAN exposure. Using the same model, Blask and George Brainard of Thomas Jefferson University have begun conducting pilot studies of the effects of melatonin and LAN on human prostate cancer.

Other studies are implicating over- or underexpression of genes known to be involved in the body’s circadian clock. For instance, Stevens and colleagues at Yale including Yong Zhu found that healthy control women showed lower expression of the *CLOCK* gene than women with breast cancer.^8^ They also found that epigenetic changes—the switching on or off of genes as a result of environmental factors—may play a role. For instance, an epigenetic change called promoter methylation, which turns off expression of *CLOCK*, was associated with lower risk of breast cancer.^8^ Stevens and Zhu are now studying whether women who work night shifts exhibit lower *CLOCK* promoter methylation.

Another big question is how much of a contribution LAN makes to cancer risk. “Light at night is likely to be one of a number of factors that contributed to the increase in breast cancer over the last few decades,” says Les Reinlib, the program director who coordinates NIEHS grants related to health effects of LAN. “It seems to be significant, and if it is, then that’s something we can control.”

**Embrace the dark... for better health**

**Ways to reduce circadian disruption resulting from LAN exposure^9^–^11^**

⟫ Consider extending the dark period at night to 9 or 10 hours. Install room-darkening shades in bedrooms.
⟫ Avoid even brief light exposures. Turn off the lights, television, and computer in the bedroom when you are sleeping. Avoid watching television or working on the computer right before you shut your eyes.
⟫ If you get up in the night, forgo the usual bathroom lights for a dim red nightlight. Red light suppresses melatonin production less than other wavelengths.
⟫ Do not take melatonin tablets unless directed by a physician. The spike in circulating melatonin may actually worsen, not alleviate, circadian disruption.
